# Exposure patterns and the risk factors of Crimean Congo hemorrhagic fever virus amongst humans, livestock and selected wild animals at the human/livestock/wildlife interface in Isiolo County, upper eastern Kenya

**DOI:** 10.1371/journal.pntd.0012083

**Published:** 2024-09-13

**Authors:** Eugine Mukhaye, James M. Akoko, Richard Nyamota, Athman Mwatondo, Mathew Muturi, Daniel Nthiwa, Lynn J. Kirwa, Joel L. Bargul, Hussein M. Abkallo, Bernard Bett

**Affiliations:** 1 International Livestock Research Institute, Nairobi, Kenya; 2 Department of Biochemistry, Jomo Kenyatta University of Agriculture and Technology, Nairobi, Kenya; 3 Department of Medical Microbiology and Immunology, University of Nairobi, Nairobi, Kenya; 4 Department of Veterinary Medicine, Dahlem Research School of Biomedical Sciences (DRS), Freie Universität Berlin, Berlin, Germany; 5 Department of Biological Sciences, University of Embu, Embu, Kenya; UDLA: Universidad de Las Americas, ECUADOR

## Abstract

Crimean Congo hemorrhagic fever (CCHF) is a tick-borne zoonotic disease caused by CCHF virus (CCHFV). The disease has a complex transmission cycle that involves a wide range of hosts including mammalian and some species of birds. We implemented a sero-epidemiological study in Isiolo County, Kenya, to determine relative seroprevalences of CCHFV in humans, livestock and in wild animals. In addition, we identified subject and environment level factors that could promote exposure to CCHFV. Humans (n = 580) and livestock (n = 2,137) were recruited into the study through a multistage random sampling technique, and in addition, various species of wild animals (n = 87) were also sampled conveniently. Serum samples from all recruited humans and animals were collected and screened for CCHFV antibodies using ID Screen multispecies, double-antigen IgG enzyme-linked immunosorbent assay (ELISA). The overall anti-CCHFV IgG seroprevalences in humans, cattle, goats, sheep and camels were 7.2% [95% CI: 3.1–15.8%], 53.9% [95% CI: 30.7–50.9%], 11.6% [95% CI: 7.2–22.5%], 8.6% [95% CI: 3–14%] and 89.7% [95% CI: 78–94%], respectively. On average, the sampled wild animals had CCHFV seroprevalence of 41.0% [95% CI: 29.1–49.4%]; giraffes had the highest mean CCHF seroprevalence followed by buffaloes, while impala had very low exposure levels. Statistical analyses using mixed effects logistic regression models showed that CCHFV exposure in humans was significantly associated with male gender, being over 30 years of age and belonging to a household with a seropositive herd. In livestock, a combination of animal- and environment level factors including older animals, being in an area with high normalized difference vegetation index (NDVI) and high vapour pressure deficit were significantly associated with CCHFV infection. Age, sex and species of wild animals were considered as the key risk factors in the analysis, but none of these variables was significant (P-value = 0.891, 0.401 and 0.664, respectively). Additionally, RT-qPCR analysis revealed the presence of CCHFV RNA in camels (30%), cattle (14.3%), and goats (3.8%), but not in humans, sheep, or wild animals. This study demonstrates that environmental factors, such as NDVI and vapor pressure deficit, affect CCHFV exposure in livestock, while the presence of infected livestock is the key determinant of human exposure at the household level. These findings underscore the importance of using One Health approaches to control the disease in human-livestock-wildlife interfaces. For instance, the existing CCHF surveillance measures could be enhanced by incorporating algorithms that simulate disease risk based on the environmental factors identified in the study. Additionally, tick control in livestock, such as the use of acaricides, could reduce CCHFV exposure in livestock and, consequently, in humans.

## Author summary

Our study focused on understanding the prevalence and risk factors associated with Crimean Congo hemorrhagic fever virus (CCHFV) exposure among humans, livestock, and wild animals in Isiolo County, Kenya. Through a comprehensive sero-epidemiological investigation and a confirmatory test using RT-qPCR, we found varying seroprevalences across different species, with camels exhibiting the highest prevalence of the various livestock species sampled. Wild animals, notably giraffes, also displayed significant seroprevalences. Factors such as gender, age, and herd seropositivity were identified as significant contributors to CCHFV exposure among humans, while environmental factors like vegetation indices and vapor pressure influenced livestock exposure.

Our findings underscore the intricate interplay between human, animal, and environmental factors in CCHFV transmission dynamics. By elucidating these factors, we provide crucial insights for policymakers to develop targeted interventions and surveillance strategies, emphasizing the importance of a One-Health approach. Implementing control measures in livestock, such as acaricide application, could effectively mitigate CCHFV transmission to humans. Additionally, the use of personal protective equipment (PPE) and workplace education for animal related professions are crucial for preventing human infections.

## 1. Introduction

Crimean Congo hemorrhagic fever (CCHF) is a zoonotic disease that is caused by tick-borne CCHF virus (CCHFV) [1]. The CCHFV is a segmented, single-stranded RNA virus of the order Bunyavirales, family Nairoviridae, and genus Orthonairovirus [2].CCHF is endemic in parts of Africa, the Middle East, Eastern Europe, Southern Europe and Asia [3]. Ticks are the primary reservoirs of CCHFV, while domestic animals, wild animals, and some avian species act as maintenance hosts and amplifiers of the virus, thus supporting its transmission [4,5]

Humans can be infected either through bites of infected ticks or via contact to the CCHF-infected animal tissues. In addition, the possibility of human-to-human infections has been reported [6]. Full-blown haemorrhagic CCHF may manifest at least four different exposure phases, namely, incubation, pre-hemorrhagic, hemorrhagic, and recuperation phases [7–9]. In most people, the disease manifests as a transient febrile illness with symptoms such as nausea, myalgia, photophobia, chills, fever, and a severe headache [10]. A small fraction of individuals may experience a more severe haemorrhagic syndrome when exposed to the virus after 5 to 14 days. This could be due to differences in the mode of exposure, as a tick bites are considered to provide a bigger infectious dose of the virus compared to that from contact with tissues from an infected animal [10].

CCHF is a significant public health threat due to its high case-fatality rate in humans, which can range from 3% to 50% depending on the level of medical care provided [6]. The risk of CCHF is further exacerbated by the (a) lack of an efficacious vaccine at present due to lack of a worldwide representative of CCHFV genotypes (b) evolution of the virus in the viral targets, which may neutralize the efficacies of available vaccines [11], (c) the absence of susceptible animal models for replicating the pathology of the virus typically encountered in humans and (d) the inadequate medication choices for the patients (with ribavirin, a broad anti-RNA virus inhibitor being the only approved treatment) [12].

CCHFV is transmitted to animals through tick bites during their blood meal. Although livestock infections with CCHFV can cause trade restrictions during outbreaks, the economic impact is relatively minor compared to other livestock diseases [13].

It also poses health risks to the value chain actors in livestock marketing chains (meat processing workers, traders, retailers, consumers) since they may be exposed to the CCHFV via handling of infected livestock tissues and/or acquire the disease through infected tick bites as host-attached ticks would be transported to the livestock market. The primary health risk to humans, however, comes from tick bites rather than direct contact with livestock blood.

CCHFV is endemic in many pastoral rangelands where people, livestock and wild animals share common geographical locations [14]. This interface brings together wild animals, livestock species, diverse species of ticks, and also humans, which provides the necessary conditions for active transmission of CCHFV, primarily through ticks that feed on both infected and uninfected animals. Animals provide blood meals for these ticks, facilitating the spread of the virus [15].

While animals are frequently exposed to CCHFV if ecological conditions favor tick populations, this does not necessarily correlate to high human exposure. Although high seroprevalence in animals indicates a significant presence of infectious ticks, human exposure to CCHFV is influenced by their livelihood practices. In a study that was conducted in the Maasai Mara ecosystem, Nanyuki, and the Ol Pejeta Conservancy in Kenya, a mean CCHFV seroprevalence of 31.5% in cattle, sheep, and goats was estimated [14]. Another study that was implemented in the same year in Lake Nakuru National Park, Solio Conservancy, Maasai Mara ecosystem, Meru National Park, and Ol Pejeta Conservancy reported CCHFV seroprevalences of 75.3% and 28.1% in buffalo and cattle, respectively [16]. Similarly, a mean CCHFV seroprevalence of 5.3% (n = 1958) was reported in cattle, sheep, and goats that were sampled in western Kenya [17], while another study that sampled animals from slaughterhouses in Nairobi, Kiambu, and Murang’sa Counties reported a seroprevalence of 4.2% (n = 2330) [13]. From these studies, the mean CCHFV seroprevalence in livestock ranges from 4.2% to 31.5% in Kenya. In humans, the mean seroprevalences that have been reported ranged from 1.9% to 5.9% [18,19].

Our study explored CCHFV seroprevalence in humans, livestock species, and wild animals among the largely pastoral community in Garbatulla subcounty, Isiolo, Kenya. A few wild animals were sampled in Ngare Mara ward, Isiolo, Kenya. The design used in this work fostered the community and public engagement approach as it brought together the professionals from the public and animal health sectors, and the local communities to address the CCHF burden. The study also utilized several environment data including climatic, topographic and geological data while investigating risk factors that influence the spatial distribution of the disease.

## 2. Materials and methods

### 2.1. Ethics statement

The study received ethical approval from the Institutional Research Ethics Committee of the International Livestock Research Institute (Reference number: ILRI-IREC2020-07). The approval for the use of animals in the study was provided by ILRI’s Institutional Animal Use and Care Committee (ILRI-IACUC 2021–18). Prior to sampling, written consents were obtained from livestock owners and household heads. For individuals below 18 years of age, written permission was acquired from their parents or legal guardians. Adults aged 18 and above provided written consent before being sampled. All aspects of human and animal sampling, as well as data collection, adhered strictly to the standard operating procedures and guidelines outlined in the ethical approval. The wild animal samples were collected in collaboration with the Kenya Wildlife Service (KWS) during their routine surveillance and animal translocation activities and as such, no ethical approval was required for this component of work.

### 2.2. The study site

**Fig 1:**
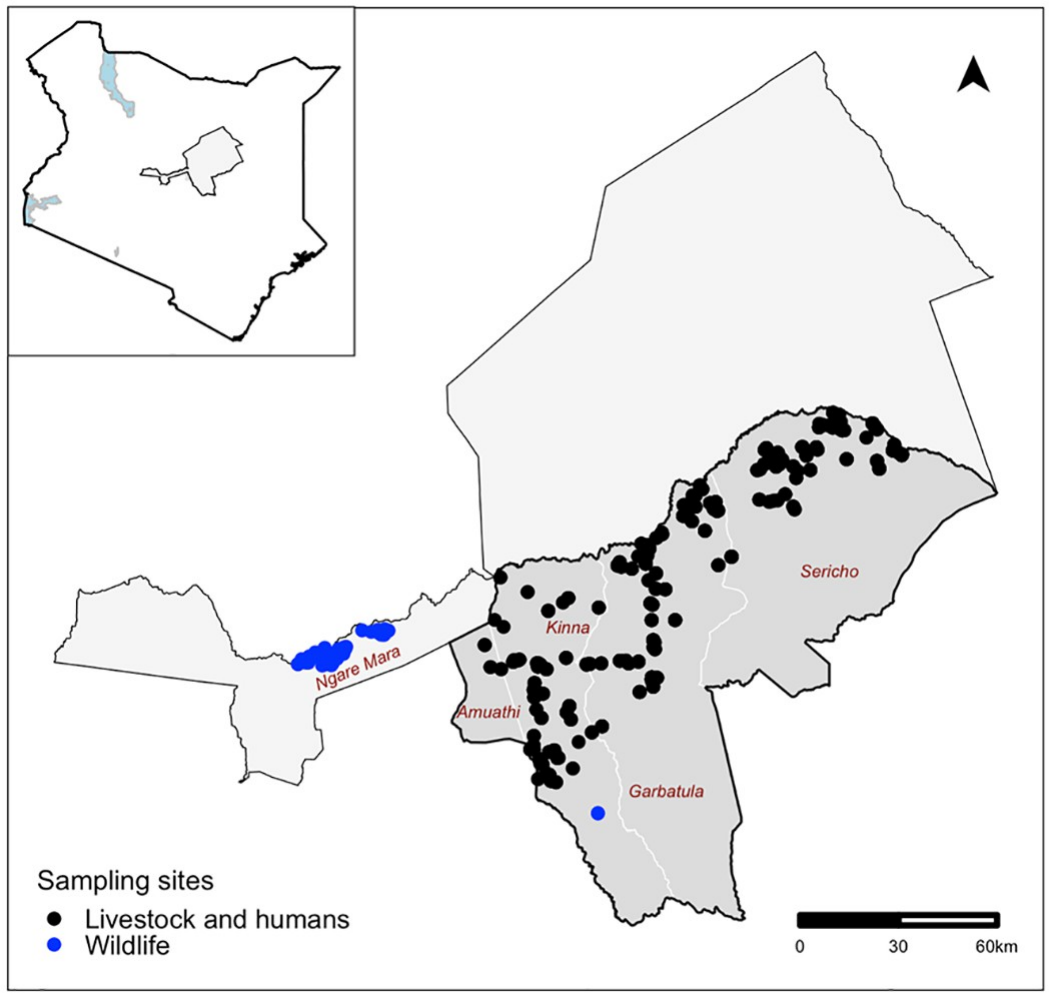
A map showing the sampling sites in Isiolo County, Kenya. The black dots represent the locations where livestock and human samples were collected from, while the blue dots represent areas where wild animals were sampled. The shapefile used to create the map was obtained from: https://gadm.org/download_country.html.

The study was carried out in Isiolo County, upper eastern Kenya (Fig 1). The area falls in an arid to semi-arid ecological zone and is inhabited by the Borana pastoralists. The community keeps cattle, sheep, goats, and camels, which are often grazed in open fields where several species of wild animals that stray from the two neighboring national parks—namely the Meru National Park in the southeast and Samburu National Park in the northwest—use as their migratory corridors. These parks were used in the study for opportunistic wild animals sampling.

The study area covered five administrative Wards in a south-west/north-east orientation (Fig 1). This belt was specifically curved out for the study since it represents a unique ecological gradient with the western side of the belt lying at an altitude of about 900 m above sea level and recording mean temperatures of 24°C, while its eastern side has a lower altitude of about 450m above sea level and higher mean temperatures of 30°C.

### 2.3. The study design

The study utilized a cross-sectional design, with random sampling procedures being used to identify human and animal subjects that were recruited for sampling. The study was carried out between July and August 2021. The number of subjects that were needed was calculated using the standard formular for determining a sample size for estimating a population proportion, i.e., *n* = *Z*^2^.*p*.(1 − *p*)/*d*^2^ [20]. In order to obtain a conservative estimate, a *priori* CCHFV seroprevalence in all host types, represented by p in the formula, was assumed to be 50%. *Z* represented a z-score from the standard normal distribution that corresponds to 95% confidence (1.96), and *d* stood for the precision of the seroprevalence estimate, which was assumed to be 5%. A naïve sample size estimate of 384 subjects from each group was produced using these parameter assumptions.

Given that humans and livestock clustered by households, grazing units, and ecological zones, it was expected that CCHFV seropositivity levels at the subject level would be correlated at each of these levels of aggregation. For simplicity, the study considered the household as the only level of aggregation, as the CCHFV seropositivity data at this level were expected to have the highest intra-cluster correlation coefficient. To account for this challenge, the naïve sample size for humans and livestock was amplified by a design effect. The design effect (Deff) was estimated using the formula *Deff* = 1 + (*b*−1)*ρ*, where b was the number of projected samples per herd/household and p was the intra-cluster correlation coefficient (ICC), which measures the rate of homogeneity. In both humans and livestock, a conservative ICC value of 0.05 was used based on the principle of using a cautious estimate when no information on the magnitude of the intra-cluster correlation coefficient at a cluster level is available [21]. A total of 242 households were therefore needed for the study, assuming that up to five (5) people and 20 animals would be selected from each household. This resulted in a design effect of 1.95 and an adjusted sample size of 749 for livestock. In humans sample size estimation, a design effect of 1.2 was used yielding a minimum sample size of 461 humans.

The requirement for the minimum sample size was not enforced for wild animals because only 87 animals could be sampled in the area.

### 2.4. Sample collection, handling, and transportation

Random Geographical Coordinates (RGC) that covered the study area were generated using the QGIS software and used to select households for sampling. A household that was closest to a given RGC was selected for sampling. A household was included in the study if it had (i) livestock, (ii) pastoralism as the main livelihood activity, (iii) at least one adult (of 18 years or older) who could provide informed consent and (iv) the head of the household provided consent for their enrollment.

A pretested questionnaire was used to collect background data of each household and subjects sampled. The questionnaire was administered using Open Data Kit (ODK) database systems. Baseline data regarding the coordinates of the household; number of animals kept; species, age, sex and body condition scores of the animals sampled; contacts for the household head; age and gender of the human subjects (pastoralists) sampled and other potential risk factors for CCHF infection were collected.

Five animals per livestock species (including cattle, camels, sheep and goats) that were kept in selected herds were identified for sampling using a systematic random sampling procedure. The criteria that were used to select livestock herds and animals for inclusion in the study included no recent history of any vaccination, animals without any discernible clinical syndrome and provision of informed consents from the livestock owners. Veterinarians from the Department of Veterinary Services, Isiolo County, were engaged for blood sampling. They collected 10 ml of venous blood from the jugular veins from each recruited animal.

Consenting participants, except children under the age of two years, were enrolled in the study and sampled after providing written consents. Parents or guardians provided written consent for children that were under the age of 18 years. Blood samples were obtained from these subjects by qualified and registered nurses from the Ministry of Health. They used non-heparinized vacutainer tubes to collect up to 5 ml of blood from the median cubital vein of the left hand from each subject.

The samples obtained from humans and livestock were promptly preserved and transported to a nearby laboratory in specialized cool boxes that kept temperatures between 4–8°C. Upon arrival at the local laboratory the samples were centrifuged at 2500 *×g* for 15 minutes to extract the serum. The extracted serum samples were then put into cryovial tubes with distinctive barcode labels. Subsequently, they were stored and transported at −20°C using a motorized freezer. The samples were also transported in this freezer to the International Livestock Research Institute (ILRI, Nairobi). At ILRI, the samples were transferred to a −20°C freezer where they were kept until they were analyzed.

The wild animals sampling surveys targeted various wild animals’ species such as giraffe, buffaloes, zebra, waterbuck, oryx, impala and warthog. Animal trapping, restraining and sampling was done by officers from the veterinary department at the KWS. All the animals were captured by chemical immobilization, except warthogs that were physically captured.

Chemical immobilant comprised a combination of an opioid Etorphine 9.8 mg/mL and a tranquilizer Azaperone 100 mg/ml. This was used to dart and immobilize the target animals. Blood samples were collected from tranquilized animals using a 10ml plain vacutainer tube from the jugular vein. After sampling, the opioid antagonists—Naltrexone 50 mg/ml or Diprenophine 12 mg/ml—were used to reverse the general anesthesia. Animals were then allowed to escape as soon as they were able to wake up. Warthogs were gently nudged into linear nets for capture using the field vehicles, then physically restrained before the collection of up to 10 ml of blood from the jugular vein using plain vacutainer tubes.

The procedure for sample storage, transportation, centrifugation, labeling and processing for all the wild animals’ samples was similar to that used for the livestock and humans samples described above.

### 2.5. Serological assay

CCHF double-antigen and multispecies ELISA Kit (ID Screen; IDvet, Grabels, France) was used to screen the serum samples collected in the study. As per the manufacturer’s instructions, the kit is 99.8% sensitive and 100% specific. Importantly, there is proven absence of cross-reactions with other nairoviruses such as Hazara virus, Dugbe virus, and Nairobi Sheep Disease Virus [22].

Fifty microliters of the ELISA kit dilution buffer was added to 96-well microplates wells pre-coated with CCHFV recombinant N protein before 30 μl of each test sample or control were added to the specified test and control wells. The plate was then wrapped and incubated at 25°C for 45 minutes. Each test well received 50 μl of the reconstituted nucleoprotein-Horseradish Peroxidase conjugate following a washing procedure, and each well then underwent a 30-minute incubation at 25°C. Following this, 100 μl of the substrate was added after a second washing process, and the mixture was incubated at 25°C for 15 min. To halt the reaction, 100 μl of stop solution was added to ach well.

A spectrophotometer set to 450 nm was used to determine the optical density value (OD) for each test sample or the control. The OD obtained for the tested sample was then divided by the test’s positive control and multiplied by 100 to determine the sample-to-positive control ratio percent (S/P%). The S/P% was utilized to interpret the results; serum with a S/P% of less than or equal to 30% was regarded negative, while serum with a S/P% of more than 30% was considered to be positive [23].

Data generated from the serological assays were entered into a database that was designed using Microsoft Excel version 2018 (IBM, California).

### 2.6. Molecular assays

Viral RNA was extracted from a 300 μl aliquot of serum pre-treated with proteinase K using the TANBead automated magnetic bead extraction technology, TANBead blood RNA kit (621 series) (TANBead, Taiwan) using the manufacturer’s instruction.

Total RNA extracted for the viral S segment was tested using a real-time reverse transcription PCR (qRT-PCR) assay [24] This assay was performed in wells duplicate of 96-well plates using the EXPRESS One-Step Superscript qRT-PCR kit (Cat No. 11791–200, ThermoFisher, France). A 5 μl of RNA sample and 25 μl reactions run were used on the QuantStudio 5 Real-Time PCR System (Thermo Scientific, Waltham, MA, USA) according to the manufacturer’s instructions. The reaction was performed at 50°C for 15 min; 95°C for 2 min and 40 cycles of 95°C for 15s, 60°C for 1min. The fluorescence data were assembled and measured after each elongation step. The positive and negative control were included in all reactions.

### 2.7. Statistical analysis

#### 2.7.1. Data preparation

Three types of data were collected in this study; these included the field datasets that were collected using questionnaires, the laboratory data that characterized each subject based on their CCHFV serological status, and environment data such as temperature, rainfall, humidity and normalized difference vegetation indices—which were obtained from on-line databases. The environment data were downloaded as raster (tif) files and clipped using the study area’s shapefile. The S1 Table lists these data sets as variables that were used in the analysis. All the analyses were conducted in R version 4.2.2.

The first step in the analysis was to merge all the data sets by host type. The spatial coordinates collected during sampling were used to extract the environment data layers. All the humans and animals sampled in the same sampling points therefore had similar values for each environment layer. Vapor pressure deficit (haP) was generated as an additional environment variable using temperature and humidity values following the algorithms described by [25].

#### 2.7.2. Descriptive analyses

Descriptive analyses were conducted using the Desctools package (https://cran.r-project.org/package=DescTools). These analyses generated CCHFV seroprevalences with their 95% confidence intervals (CI), and stratified these by the independent factors determined in the field. The independent factors considered for the human data included age, gender, and the administrative ward where the subject belonged, while those that were considered for livestock included species, sex, age and ward. Given that there were few records for the wild animals, wild animals data were collapsed into two categories. The first included all the records from bovidae (buffalo, oryx, waterbuck and impala) while the others included non-bovidae (giraffe, zebra and warthog). Fisher’s exact tests were used to estimate unadjusted associations between the independent factors and CCHFV seroprevalence.

#### 2.7.3. Statistical modelling

Univariable and multivariable modelling was conducted to identify risk factors that influenced CCHFV seroprevalence. The univariable analysis were conducted using Generalized Linear Model (GLM). This was aimed to explore unadjusted association between the various independent factors considered and the CCHFV seroprevalence. For the human data, univariable analysis used gender, age group, ward, elevation (dem), Normalized Difference Vegetation Index (NDVI), aridity, mean annual rainfall (mrain), CCHFV seroprevalence in livestock, temperature, humidity and Vapor Pressure Deficit (VPD). Gender variable was coded as male or female and age group included 2–14 years, 15–29 years, 30–44 years and over 44 years. The subjects sampled came from three wards and the levels used in the analysis were Sericho, Garbatulla and Kinna. The rest of the factors were used as continuous variables. CCHFV seroprevalence in livestock at the household level was estimated and merged with the human data so as to investigate whether there was any significant association between the human and livestock exposure. We assumed that the effective human and livestock interactions and contacts occurred at this level.

The analysis of the livestock data used similar data as that of humans. However, age groups in livestock were classified into calf/kid/lamb, young adult and adults. Calves/kids/lambs included animals that had not been weaned and were therefore being kept around the homesteads throughout the day. Young adults included animals that had not matured but had been allowed to go out for grazing with the mature animals. The species used in the analyses included goats, sheep, cattle and camels. For the wild animals’ data, modelling used age, species and the sex of an animal as independent factors. Age was classified as sub-adult and adults, while species were bovidae and non-bovidae.

Multivariable analyses were conducted using a generalized linear mixed model (GLMM) to account for potential clustering of data at the household level. All the continuous variables were assessed for their linearity assumption. Two-way interaction terms between independent variables were also evaluated.

## 3. Results

### 3.1. Serological assay

The study obtained a total of 580, 2,137 and 87 samples from humans, livestock and wild animals. The overall seroprevalence of CCHFV in humans, livestock and wild animals was 7.2% (95% CI: 3.78–15.51%), 43.1% (95% CI: 30.7–50.3%) and 41.0% (95% CI: 35–85%), respectively. Tables 1, 2 and 3 provides descriptive analyses of these data by host group.

**Table 1:** Seroprevalence of CCHFV in humans.

Species	Variable	Level	n	Positive	% (95 CI)	P-value
Humans	Sex	Male	482	39	8.1 (6.0–10.9)	0.121
		Female	98	3	3.1 (1.0–8.6)	
	Age in years	2–14	58	1	1.7 (0.01–9.1)	0.004
		15–29	177	5	2.8 (1.2–6.4)	
		30–44	143	15	10.5 (6.5–16.6)	
		45+	202	21	10.4 (6.9–15.5)	
	Ward	Sericho	234	22	9.4 (6.3–13.8)	0.253
		Garbatulla	284	15	5.3 (3.2–8.5)	
		Kinna	82	5	6.1 (2.6–13.5)	

**Table 2:** Seroprevalence of CCHFV in livestock.

Species	Variable	Level	n	Positive	% (95 CI)	P-value
Livestock	Species	Cattle	160	100	62.5 (54.8–69.6)	0.001
		Sheep	750	66	8.8 (6.9–11.0)	
		Goats	841	96	11.4 (9.4–13.7)	
		Camels	97	87	89.6 (82.1–94.3)	
	Sex	Male	410	52	12.7 (9.8–16.3)	0.011
		Female	1358	227	16.7 (14.8–18.8)	
	Age	Adult	1514	255	16.8 (15.0–18.8)	0.007
		Young adult	207	14	6.8 (4.1–11.0)	
		Calves	47	10	21.3 (11.9–34.9)	
	Ward	Sericho	511	134	26.2 (22.5–30.2)	0.001
		Garbatulla	932	98	10.5 (8.7–12.6)	
		Kinna	325	47	14.5 (11.1–18.7)	

**Table 3:** Seroprevalence of CCHFV in wildlife.

Species	Variable	Level	n	Positive	% (95 CI)	P-value
Wildlife	Species	Giraffe	12	9	75.0 (43.0–95.0)	0.017
		Buffalo	16	10	62.5 (35.0–85.0)	
		Zebra	15	6	40.0 (16.3–68.7)	
		Warthog	15	3	20.0 (4.7–48.9)	
		Waterbuck	6	1	16.7 (0.0–64.0)	
		Oryx	18	6	33.3 (13.2–59.3)	
		Impala	3	0	0.0 (0.0–71.2)	
	Sex	Male	54	20	38.8 (27.7–53.2)	0.636
		Female	33	16	45.4 (30.3–63.3)	
	Age	Sub-adult	18	8	44.4 (27.7–70.5)	0.978
		adult	69	28	40.5 (30.4–53.3)	

### 3.2. Molecular assays

In addition to serological surveillance, RT-qPCR analysis was conducted on a few samples that were selected randomly to detect the presence of CCHFV RNA. The results are summarized in Table 4 below:

**Table 4:** RT-qPCR results showing virus detection in humans, livestock and wild animals.

Species	No. tested	No. of positives
Humans	16	0
Camels	80	24
Cattle	42	6
Goats	26	1
Sheep	16	0
Wild animals	7	0

### 3.3. Results of the univariable analyses

The univariable analyses of the human data suggest that there was a significant association between CCHFV seroprevalence and advanced age and higher livestock seroprevalence at the household level. In both cases, the odds of exposure increased linearly with age and livestock CCHFV seroprevalence. For livestock data, low mean rainfall and an increase in vapor pressure deficit were the two key environment variables that were associated with high CCHFV exposure. Similarly, animals that were sampled in Sericho also had higher CCHFV exposure than those sampled in the other wards. For wild animals’ data, none of the variables species was associated with CCHFV exposure.

### 3.4. Results from multivariable analyses

Table 5 provides the results from the multivariable analyses conducted on human, livestock and wild animal data. The results show that each model identified a unique set of variables that were identified as being significant.

**Table 5:** Mixed effects multivariable logistic regression analysis of the association between risk factors and CCHFV seropositivity in humans, livestock and wild animals.

Species	Variable	Level	OR (95% CI)	P-value
Humans	Gender	Female	1.00	
		Male	3.38 (1.00–11.42)	0.014
	Age group	2–14	0.59 (0.07–5.22)	0.641
		14–29	1.00	
		30–44	4.72 (1.66–13.44)	0.001
		45+	4.72 (1.73–12.91)	0.001
	Livestock seroprevalence (%)	<10	1.00	
		11–20	1.98 (0.79–4.94)	0.132
		21–30	1.51 (0.31–7.26)	0.614
		>30	2.36 (1.06–5.23)	0.041
Livestock	Age	Adult	1.00	
		Calf/kid/lamb	1.11 (0.44–2.81)	0.757
		Young adult	0.31 (0.16–0.60)	0.001
	NDVI	-	4.01 (1.15–13.94)	0.028
	Vapor pressure deficit	-	1.25 (1.13–1.39)	0.001
Wildlife	Species	Bovidae	1.00	
		Non-bovidae	1.23 (0.49–3.12)	0.664
	Sex	Female	1.00	
		Male	0.69 (0.28–1.66)	0.401
	Age	Adult	1.00	
		Sub-adult	1.08 (0.34–3.32)	0.891

The model fitted to the human data shows that being of a male gender was associated with significantly higher odds of CCHFV exposure compared to being of a female gender. Older people, especially those over 30 years, also had higher odds of exposure compared to younger ones. In fact, young people of 2–14 years were found to be a protective factor. Similarly, people from households that had livestock with >30% CCHFV seroprevalence had a higher risk of exposure compared to those that did not.

For the livestock data, young adult animals had significantly lower odds of CCHFV exposure compared to adults or calves/kids/lambs. Similarly, higher NDVI and vapour pressure deficit was associated with greater odds of CCHFV exposure.

### 3.5. Intracluster correlation coefficient

The intracluster correlation coefficient (ICC) was calculated to assess the degree of similarity among observations within the same cluster (household). ICC values were computed separately for the human and livestock datasets. The ICC values for CCHFV seropositivity in the human and livestock dataset were found to be higher than the conservative estimate used for sample size calculation, at 0.25 (95% CI: 0.18–0.32) and 0.29 (95% CI: 0.12–0.34) respectively.

## 4. Discussion

This study investigated CCHFV seroprevalence and factors that influence the infection patterns of the virus in humans, livestock and wild animals in an arid to semi-arid area in northern Kenya where pastoralism is the main source of livelihoods. The CCHFV seroprevalences that were estimated varied significantly between households, livestock and different wild animals’ species. Notably, the livestock and wild animals’ seroprevalences were higher than those for humans.

Sero-epidemiological studies are increasingly being used to study the burden and risk factors of a wide range of infectious agents given their ability to detect both clinical and sub-clinical infections. This is particularly relevant for CCHFV given that most of the hosts that the virus infects do not show any clinical signs. It is also suspected that many human infections that occur especially in remote locations go undetected due to poor diagnostic facilities. The data obtained from this (and similar studies) are therefore invaluable in identifying hotspots that can be targeted for intensive surveys.

The study detected a low CCHFV seroprevalence in humans. Being a male, having an advanced age (>30 years old) or belonging to a household that had livestock with a high CCHFV seroprevalence were associated with higher CCHFV seroprevalence compared to being a female, being younger or belonging to a household that had animals with lower seroprevalence. Similar findings were reported by [26,27]. The higher CCHFV seroprevalence in males could be attributed to occupational exposure given that men were more likely to run outdoor occupational activities that could increase their contact with ticks [28] or infectious animals. Males were more likely to be in charge of herding animals, trekking them to the markets or slaughtering animals. With respect to age, a high CCHFV seroprevalence was observed in the subjects that had more than 30 years. A similar finding was reported in South Africa where older farmers had higher prevalence of CCHF IgG antibodies compared to younger ones [29]. The higher CCHFV seroprevalence in older people could be associated with cumulative exposures over time. This is a common finding in many sero-epidemiological investigations [30].

The study found a positive association between human exposure and livestock CCHFV seroprevalence at the household level particularly if a livestock herd had high CCHFV seroprevalence. This observation suggests that humans likely acquired CCHFV infections primarily through bites from ticks feeding on their livestock. Although less frequent, direct infection from infected blood during livestock slaughtering can also occur. However, this direct transmission route is limited due to the short viremia period in livestock and the infrequency of slaughtering events in extensive pastoralist systems. This observation indicates that a significant number of people acquired CCHFV infections either directly or indirectly from their own livestock [1]. The significant association between livestock and human CCHFV infections highlights the potential impact of controlling the disease in livestock, for example through the use of acaricides, on human exposure. This finding also highlights the need to account for practices that are used to control ticks in livestock while investigating CCHFV seroprevalence in humans.

There was an enormous variability on CCHFV seroprevalences between livestock species. Notably, camels had the highest seroprevalence that peaked at 89.6%. Similar exposure patterns have been described by [31] in southern Tunisia. The higher prevalence of CCHFV observed in camels compared to other animals could be attributed to several factors, including the higher prevalence of tick vectors in regions where camels are commonly grazed. Additionally, variations in the use of acaricides, differences in tick species that infest camels, and specific ecological conditions of these regions might contribute to this increased prevalence. Further studies are needed to explore these factors in more detail to understand the reasons behind the higher seroprevalence in camels. The high seroprevalence in camels could suggest that this host plays an important role in the transmission of CCHFV [32]. However, several countries with a history of repeated human infections, such as Uganda, do not raise camels in regions that have had outbreaks. Some of the risk factors that have been identified in those studies include livestock (cattle) farming, collecting ticks and age of the subject [1].

The analyses further identified significant differences on CCHFV between age groups in livestock; mature animals and calves/kids/lambs had higher seroprevalences than the young adults. This finding is consistent with the work of [5], and it can be supported by the fact that older animals might have had repeated exposures over time. Sero-epidemiological data, as illustrated above, include cumulative exposures that an animal might have experienced throughout its lifetime as the outcome. Adult animals might have therefore had higher chances of being infected compared to young adults. Calves/kids/lambs however present a different exposure pattern—the high exposure levels in this age group could be associated with the presence of maternal antibodies in their system [14].

**Fig 2:**
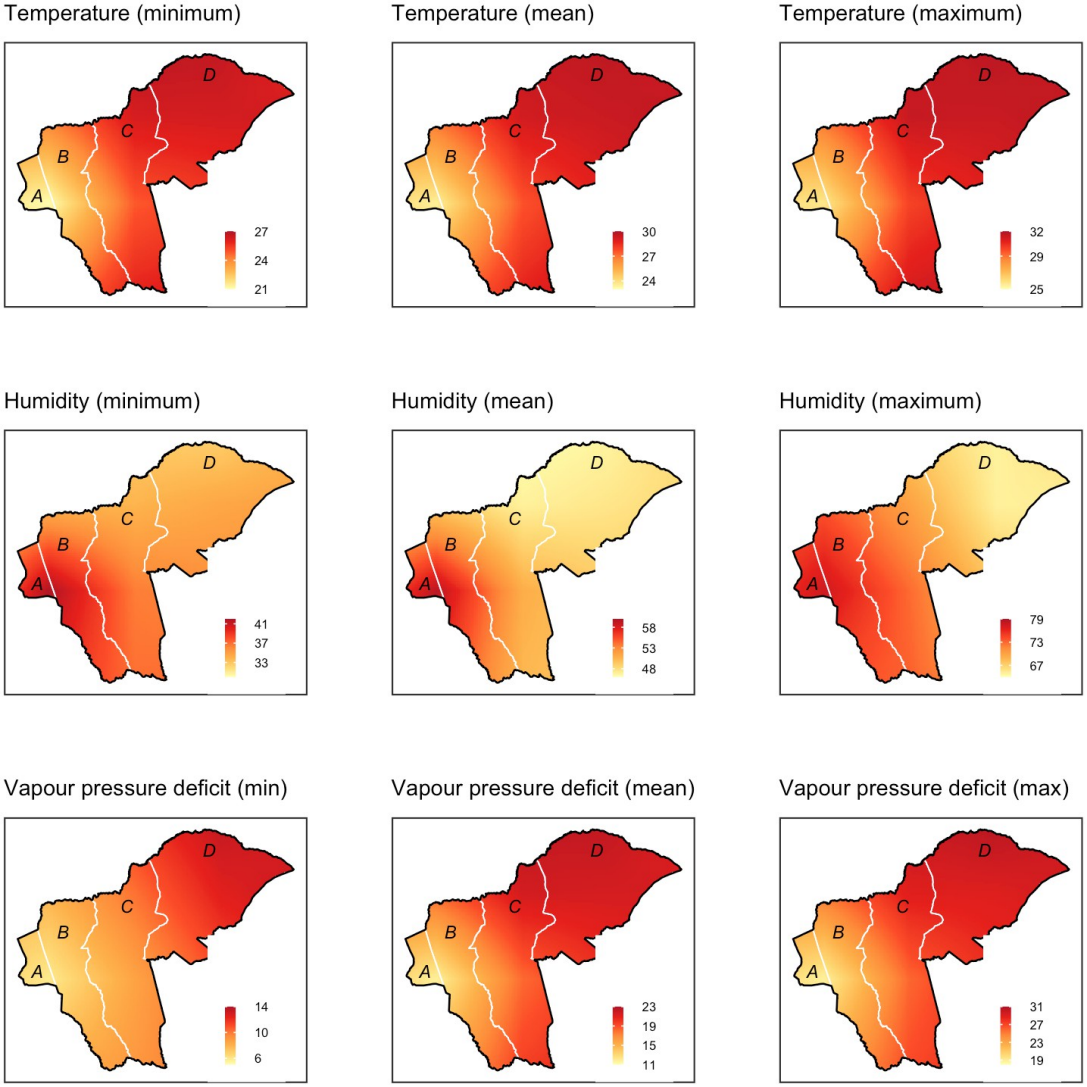
Maps showing estimates of temperature, humidity and vapor pressure deficit in the study area based on the data obtained from ECMWF. The letters A–D stand for the names of the wards which are A-Amwathi, B- Kinna, C- Garbatulla and D-Sericho. The map was prepared by Max Korir using QGIS version 3.36.3. The geoclimatic zone datasets were retrieved from https://www.ecmwf.int/.

The CCHFV exposure patterns observed in livestock differed slightly from those of humans in as far as they identified a few environmental variables that were significant. Previous studies by [33] in Kenya and [34] in Senegal identified geographical factors as being important in defining the hotspots for the disease. During the elementary stages of the current analysis (univariable analysis), wards (administrative units) had a significant association with the outcome. Sericho ward exhibited the highest seroprevalence compared to Kinna and Garbatulla. On further analyses, it became apparent that Sericho also had the highest minimum vapor pressure deficit compared to the other wards (Fig 2). The vapor pressure deficit also became significant in the final model, indicating that selected environmental factors played a big role in making this area suitable for the virus transmission. *Hyalomma* ticks, the main vectors of CCHFV thrive well in areas with high vapor pressure deficit [28]; this indicates that the same vector may be playing a role in the transmission of the virus in this region although more studies should be done to verify this observation.

The study also investigated several potential risk factors associated with the prevalence of CCHFV in wild animals. None of the factors investigated using multivariable models were significant. A previous study by [16] did not also find significant association between sex and CCHFV prevalence in wild animals. Similarly, [5] did not find any association between CCHFV exposure and age in wild animals. Nonetheless, it is important to recognize that age-related differences in susceptibility to CCHFV might vary across different wildlife species or populations, warranting additional research to better understand age-related dynamics. The differences in the infection patterns observed in wild animals and livestock data in the current study could be attributed by several factors in addition to the sampling techniques used. These factors include the ecological differences between the sampling locations, the behavior and habitat preferences of the different species, and potential variations in tick infestation rates. Livestock sampling was more structured and enabled the recruitment of animals from at least three age groups. The approach used for sampling wild animals did not provide a reliable way of recruiting animals of various age groups. The locations used for wild animals sampling were also clustered in the two national parks that were found in the extreme western region of the study area, potentially affecting the representativeness of the data. Apart from the variability of CCHFV by species, no other variable was found to be significant.

It is also important to note the role of livestock-wild animals’ interaction in the spread of CCHFV. The grazing requirements of the Bovidae species tend to overlap with domesticated livestock especially the cattle, which facilitates cross-transmission of several infectious diseases and ectoparasites [16]. In this study, the average prevalence of CCHFV in wild animals’ bovidae species was higher compared to livestock’s average seropositivity, and because of the grazing overlap, wild animals likely serve as a source of infection to cattle. The role of wild animals, especially the bovidae species, on the epidemiology of CCHFV goes beyond its host role and includes a potential source of direct human transmission. The illegal hunting, slaughter, and consumption of buffalo in Kenya [35] may cause CCHFV transmission to the hunters as they handle infected blood [34].

The analysis also identified normalized difference vegetation index (NDVI) as being a significant predictor on CCHFV exposure in livestock. The NDVI, which indicates vegetation health and density, has been shown to be significantly associated with the prevalence of CCHFV in both humans and livestock. The study reveals that areas with a higher NDVI (mean = 0.59, SD = 0.18) have a significantly higher number of positive CCHFV samples in both humans and livestock populations compared to areas with lower NDVI values (mean = 0.56, SD = 0.18) (T-test, P < 0.05, t-value = 42.02). This suggests that regions with healthier and denser vegetation might provide a more suitable environment for ticks, contributing to an increased risk of CCHFV transmission to both humans and livestock. This finding corroborates the work of [33], which identified specific geographic areas as potential risk zones for CCHFV outbreaks.

The results of the environmental factors analysis highlight the significance of certain ecological parameters in influencing the prevalence and transmission of Crimean-Congo Hemorrhagic Fever Virus (CCHFV) to both humans and livestock. In regions where, ecological conditions favor the widespread presence of infected ticks, such as (high NDVI), preventing exposure in animals may prove challenging. Moreover, early infection in animals could contribute to the establishment of endemicity and the development of immunity, potentially reducing the risk of transmission to humans. Understanding these environmental factors is vital for developing targeted and effective strategies to mitigate the risk of CCHFV transmission and enhance disease surveillance and prevention in both human and livestock populations.

The molecular assay results using RT-qPCR further confirmed the presence of CCHFV RNA in the sampled populations. Specifically, CCHFV RNA was detected in 30% of camels, 14.3% of cattle, and 3.8% of goats. No viral RNA was detected in humans, sheep, or wildlife samples. These findings align with the serological data, suggesting that camels are a significant host for the virus, followed by cattle and goats. The absence of viral RNA in human samples corroborates the low seroprevalence observed and indicates that while exposure occurs, active infection might be less common or transient. The lack of detection in wildlife could be due to the limited number of animals that were used. These results underscore the importance of integrating molecular techniques with serological surveys to provide a comprehensive understanding of CCHFV epidemiology.

Occupational hazards play a significant role in the spread and contamination of Crimean-Congo Hemorrhagic Fever Virus (CCHFV). Professions such as veterinarians, abattoir workers, and other individuals involved in animal handling are at higher risk due to their increased exposure to potentially infected animals and ticks. Implementing personal protective equipment (PPE) and enhancing workplace education are crucial measures for reducing these occupational risks. Furthermore, a collaborative One Health approach, integrating human, animal, and environmental health sectors, is essential for effective disease control. Establishing permanent links between these sectors through joint surveillance systems and community-based reporting of symptoms can enhance early detection and response to CCHFV outbreaks. Such integrated strategies can lead to more comprehensive and sustainable control measures, ultimately reducing the transmission of CCHFV among humans and animals. The existing surveillances systems for the disease can be enhanced by integrating algorithms that predict its risk based on the environment factors identified. One Health approaches can also be used to evaluate the impact of tick control measures on the burden of the disease in animals and humans.

The intracluster correlation coefficient is critical in our study as it provides a quantifiable measure of the similarity among observations within the same cluster. The ICC values we got for each group 0.25 (95% CI: 0.18–0.32) for humans and 0.29 (95% CI: 0.12–0.34) for livestock provide insight into the degree to which CCHFV seropositivity is clustered within households. This information is crucial because a high ICC indicates that individuals within the same household are more similar in terms of CCHFV seropositivity, suggesting a substantial influence of local factors or shared characteristics. Understanding this clustering effect is vital for targeted interventions, as interventions tailored to specific regions may yield more pronounced effects. Second, accurate estimate of the model parameters and reliable inference depend on the analysis taking clustering into account. If clustering is not accounted for, variance measures for parameters derived would be underestimated. This would increase the risk of type I errors in inferential analyses.

Despite the higher ICC values observed in this study, the sample size was adequate for the inference analysis, as the study design had incorporated a cautious estimate of the ICC, ensuring the robustness of the results. This underscores the importance of considering potential clustering effects in epidemiological studies to maintain the validity of statistical inferences.

While this study has yielded important insights, there are several limitations that warrant consideration in future research. Firstly, the observed higher human exposure in areas with increased animal seropositivity may not only indicate direct or indirect transmission from animals but could also reflect a higher prevalence of tick vectors in those areas. Unfortunately, our study did not collect specific data on tick distribution and prevalence, nor did it assess the use of acaricides, which could significantly impact tick populations and, consequently, the risk of CCHFV transmission. Future research studies should include detailed tick surveillance and acaricide usage data to better understand these dynamics. Furthermore, the absence of comprehensive data on specific tick species hinders a thorough examination of tick-vector competence and the prevailing CCHFV strains within the region. Integrating vector surveillance and viral genotyping into future investigations is crucial for advancing our comprehension of the virus’s transmission dynamics.

Secondly, the limited number of wild animals’ samples included in this study may restrict the breadth of our understanding of CCHFV dynamics in the ecosystem.

## Conclusion and recommendations

However, the study’s results have crucial implications for public health. The identification of high-risk areas for CCHFV transmission can inform targeted prevention and control strategies. Increasing public awareness about CCHFV transmission, especially among high-risk populations and healthcare workers, is essential for early detection and prompt management of cases. Furthermore, close collaboration between the human and animal health sectors is crucial for effective disease surveillance, outbreak response, and preventive measures.

In conclusion, this study showed that CCHFV is prevalent in the study area even though no clinical events of the disease have been reported previously. While the virus is prevalent in livestock and wild animals, these animals typically develop immunity after a short period of viremia. Therefore, they do not serve as long-term reservoirs for the virus but rather as transient hosts for the tick vectors. The study also identified NDVI and VPD as being important environment risk factors. Both of these are known to facilitate the development of ticks.

## Supporting information

S1 Table The environment variables used in the study, including their units of measurement, source and the primary resolution. (DOCX)

S1 Dataset All the dataset that was used in this study, including human, livestock and wildlife data. (ZIP)
