# Supplementary material for: Exposure patterns and the risk factors of Crimean Congo hemorrhagic fever virus amongst humans, livestock and selected wild animals at the human/livestock/wildlife interface in Isiolo County, upper eastern Kenya
Source: PLoS Negl Trop Dis. 2024 Sep 13;18(9):e0012083. doi: 10.1371/journal.pntd.0012083 (PMC11423962; doi:10.1371/journal.pntd.0012083)
Supplement: S1 Table — (DOCX) [file pntd.0012083.s001.docx]

**S1 Table.** A table showing the environment variables used in the study, including their units of measurement, source and the primary resolution

| Dataset | Units | Source | Spatial resolution |
| --- | --- | --- | --- |
| NDVI | Index | Google Earth Engine | 30 m |
| LST | °C | Google Earth Engine | 30 m |
| Land use/land cover |  | Google Earth Engine | 30 m |
| Rainfall | Millimetres (mL) | <https://data.chc.ucsb.edu/products/CHIRPS-2.0/africa_3-monthly/tifs/>) | 5 km |
| Elevation | Meters | <https://www.diva-gis.org/gdata>). | 30 m |
| Temperature | K | ECMWF | 11 km |
| Humidity | % | ECMWF | 11 km |
